# Benchmarking Open-Source Pathology Foundation Models for Breast Cancer Biomarker Prediction from H&E Whole-Slide Images

**DOI:** 10.3390/cancers18152475

**Published:** 2026-08-01

**Authors:** Samir Atiya, Jiayou Liang, Kwaku Ofori-Atta, Michelle Peng, Huili Wang, Yifei Zhou, Ankush Patel, Mary Edgertion, Junhan Zhao, Utku Pamuksuz

**Affiliations:** 1 Department of Pathology, University of Chicago Medical Center, Chicago, IL 60637, USA; 2 Biological Science Division, University of Chicago, Chicago, IL 60637, USA; 3 Trillium Health Partners, Mississauga, ON L5B 1B8, Canada; 4 Data Science Institute, University of Chicago, Chicago, IL 60615, USA; jiayouliang@uchicago.edu (J.L.); kwaku@uchicago.edu (K.O.-A.); michellexpeng@uchicago.edu (M.P.); ankush@digitalpathomics.com (A.P.);; 5 Department of Pathology, Ohio State University, Colombus, OH 43210, USA; 6 Department of Pathology, University of Nebraska Medical Center, Omaha, NE 68106, USA; 7 Department of Pediatrics, University of Chicago Medical Center, Chicago, IL 60637, USA; 8 Department of Biomedical Informatics, Harvard Medical School, Boston, MA 02115, USA; 9 Department of Surgery, Massachusetts General Hospitals, Boston, MA 02114, USA

**Keywords:** digital pathology, artificial intelligence, whole slide imaging, breast cancer, molecular inference, IHC, foundation models, AUPRC

## Abstract

Background/Objectives: Breast cancer biomarker detection through immunohistochemistry (IHC) is essential for treatment planning but faces challenges including turnaround time, variability, and laboratory resource constraints. Large open-source vision-language foundation models offer a potential avenue for inferring biomarker status directly from hematoxylin-and-eosin (H&E)-stained whole-slide images (WSIs). Methods: We evaluated two open-source pathology foundation models—TITAN (Transformer-based Pathology Image and Text Alignment Network, approximately 48.5 M parameters) and CHIEF (Clinical Histopathology Imaging Evaluation Foundation Model, approximately 1.2 M parameters)—for predicting estrogen receptor (ER), progesterone receptor (PR), and human epidermal growth factor receptor 2 (HER2) status from H&E-stained breast cancer WSIs. WSI data were obtained from The Cancer Genome Atlas Breast Invasive Carcinoma collection (TCGA-BRCA) via the NCI Imaging Data Commons, with biomarker labels from the NCI Genomic Data Commons. In total, 937 cases (995 WSIs; 78.3% ER-positive) were evaluated for ER, 934 cases (992 WSIs; 68.4% PR-positive) for PR, and 646 cases (691 WSIs; 21.1% HER2-positive) for HER2. All evaluation was performed under a strict patient-level 50/25/25 split with 10 independent random partitions; metrics are reported as the mean across partitions with percentile-based 95% confidence intervals. Performance was assessed using area under the receiver operating characteristic curve (AUROC), area under the precision-recall curve (AUPRC), sensitivity, specificity, and positive predictive value (PPV). Results: TITAN and CHIEF achieved comparable performance for ER (TITAN AUROC: 0.885 [95% CI: 0.848, 0.921], AUPRC: 0.954 [0.940, 0.964]; CHIEF AUROC: 0.877 [0.831, 0.914], AUPRC: 0.955 [0.938, 0.969]) and PR (TITAN AUROC: 0.799, AUPRC: 0.868; CHIEF AUROC: 0.791, AUPRC: 0.864). At the default 0.5 operating point, ER PPV was 0.90 and PR PPV was 0.79–0.81. For HER2, both models achieved AUROC values of 0.71–0.74 and AUPRC values of 0.41–0.45—well above the prevalence-based random baseline (approximately 0.211)—but default-threshold sensitivity was very low (approximately 0.07–0.08), reflecting class imbalance and the use of an uncalibrated default threshold rather than a categorical absence of morphologic signal. Conclusions: Under retrospective evaluation, both models demonstrate strong discriminative performance for ER and moderate performance for PR; HER2 prediction at the default operating point is limited and motivates threshold-calibration and multimodal extensions before any clinical use. AUPRC summarizes precision−recall behavior across thresholds and is distinct from threshold-specific precision (PPV); the two should be reported together for clinical-utility assessment in pathology AI. The findings are hypothesis-generating and motivate prospective external validation across independent institutional cohorts before any clinical deployment is considered.

## Simple Summary

Breast cancer treatment is guided by three molecular biomarkers—estrogen receptor (ER), progesterone receptor (PR), and HER2—typically measured by immunohistochemistry (IHC), a process that requires additional staining, time, and specialist review. Whole-slide image (WSI) foundation models are large pre-trained neural networks that summarize a digital biopsy into a compact representation, raising the possibility of inferring biomarker status directly from routinely stained H&E images. In this study, we compare two open-source pathology foundation models—TITAN and CHIEF—for ER, PR, and HER2 prediction on the publicly available TCGA-BRCA cohort, under a strict patient-level evaluation protocol with 10 random partitions and 95% confidence intervals. ER and PR predictions show robust discriminative performance under retrospective evaluation; HER2 prediction at the default decision threshold remains limited and motivates threshold-calibration and multimodal extensions. The findings are hypothesis-generating and motivate prospective external validation before any clinical use.

## 1. Introduction

Accurate diagnosis and biomarker status determination are essential for breast cancer care. In current pathology workflow, histological slides are reviewed by a pathologist to render a diagnosis. When breast cancer is identified, additional immunohistochemical (IHC) biomarkers, primarily estrogen receptor (ER), progesterone receptor (PR), and human epidermal growth factor receptor 2 (HER2), are routinely ordered [1,2,3]. These markers direct therapy selection by determining eligibility for targeted agents, risk stratification, and surveillance planning [1,4]. However, real-world variability in tissue handling, staining protocols, scanner characteristics, and interpretive thresholds introduces noise that can undermine consistency, timeliness, and equity in clinical decision-making [5]. The current shortage of skilled laboratory histotechnologists further delays case sign-out by increasing turnaround time (TAT) for biomarker testing [6,7].

Against this backdrop, pathology foundation models (FMs) have emerged as a compelling paradigm for precision oncology. By training over heterogeneous inputs—whole slide images (WSIs), IHC results, and molecular findings—FMs promise scalable inference with the flexibility of in-context learning [8,9]. Their ability to perform IHC inference in near real time offers a path to unify fragmented workflow streams, reduce reliance on external equipment and minimize laboratory staff workload [7,10]. However, enthusiasm must be matched by rigorous validation. Foundation-scale models can exhibit overconfidence, domain hallucinations, shortcut learning, and hidden biases [11,12]. Performance reported on convenience datasets rarely translates to clinical practice without systematic assessment of generalizability, calibration, and fairness across institutions, scanners, stains, and patient subgroups [13,14]. Interpretability must extend beyond saliency to faithful rationales that pathologists can interrogate, and operational constraints including latency, reproducibility, and governance must be addressed to support clinical integration [15,16].

The landscape of pathology FMs is rapidly evolving. As of 2025, a diverse ecosystem includes general vision encoders such as UNI and CONCH, multimodal models such as PLIP and BioViL, and WSI-level models such as TITAN and CHIEF [17,18,19]. Among WSI-level models—which are most relevant for slide-level biomarker inference—we selected TITAN and CHIEF for specific and complementary reasons. TITAN represents the current state-of-the-art among open-source WSI foundation models, with benchmarking studies demonstrating superiority over earlier models including UNI and CONCH across multiple computational pathology tasks [18,20]. CHIEF, by contrast, was selected because it is the smallest FM across the major open-source foundation models, requiring substantially less computational resources and offering shorter inference times [21]. This practical footprint makes CHIEF uniquely suited for integration into low-cost devices, point-of-care settings, and resource-limited healthcare environments, including the potential for embedding directly into digital microscopy systems. Evaluating both models allows us to contrast state-of-the-art performance against a pragmatically deployable alternative.

Open-source FMs are especially attractive because they enable transparent audit, reproducibility, and adaptation for local workflows [17,22]. Rather than training a bespoke model on imperfectly annotated institutional data, an approach that introduces labeling noise, requires substantial computational infrastructure and still demands rigorous external validation, we evaluate pre-trained foundation models using frozen embeddings with a lightweight linear probe (logistic regression), avoiding end-to-end fine-tuning while enabling task-specific calibration. This approach leverages large-scale pre-training while avoiding the pitfalls of training on noisy ground truth labels and provides an immediately reproducible and auditable baseline for the community.

A critical methodological contribution of this work is the systematic use of area under the precision−recall curve (AUPRC) alongside AUROC. AUROC is the dominant metric in the pathology AI literature because it measures overall discriminatory ability regardless of classification threshold and is invariant to class imbalance in terms of ranking. However, for clinical deployment decisions, AUPRC provides essential complementary information: it directly quantifies the precision−recall trade-off that clinicians face when acting on an automated biomarker call. Unlike AUROC, AUPRC heavily penalizes false positives, a critical consideration when false-positive biomarker calls lead to unnecessary, costly, or toxic therapy. Importantly, the expected AUPRC for a random classifier equals the prevalence of the positive class, so performance above that baseline is immediately interpretable in terms of clinical gain over default prediction. For HER2 with a prevalence of ~19%, an AUROC of 0.69 appears marginally functional, but an AUPRC of 0.30–0.33 reveals that two-thirds of positive predictions are incorrect—an unacceptable rate for a therapy-determining biomarker. This study, therefore, establishes reporting AUPRC alongside AUROC as a minimum standard for clinical utility assessment in pathology AI.

## 2. Materials and Methods

### 2.1. Data Source

This study utilized publicly available breast cancer WSI data from The Cancer Genome Atlas Breast Invasive Carcinoma (TCGA-BRCA) collection, accessed via the National Cancer Institute (NCI, Bethesda, MD, USA) Imaging Data Commons (IDC) [23,24]. TCGA-BRCA is a landmark multi-institutional dataset assembled by The Cancer Genome Atlas Research Network, comprising genomic, transcriptomic, proteomic, and imaging data from breast cancer cases across multiple contributing institutions in the United States [25]. Corresponding biomarker (ER, PR, HER2) ground truth labels were sourced from the NCI Genomic Data Commons (GDC), which curates and harmonizes molecular and clinical data from TCGA and related consortia [26]. We, therefore, gratefully acknowledge the original specimen collection, data curation, and deposition work of the TCGA Research Network and the Cancer Imaging Archive (TCIA).

The IDC repository contained 1098 unique cases spanning 9 disease categories; this study focused solely on ductal and lobular carcinomas. After linking WSIs with biomarker data, 937 cases with 995 WSIs were available for ER (slide-level positivity 78.3%; 779/995 positive, 216/995 negative), 934 cases with 992 WSIs for PR (slide-level positivity 68.4%; 679/992 positive, 313/992 negative), and 646 cases with 691 WSIs for HER2 (slide-level positivity 21.1%; 146/691 positive, 545/691 negative). Cases with equivocal IHC HER2 (2+) results and absent fluorescent in situ hybridization (FISH) data were excluded to ensure definitive ground truth labels, consistent with ASCO/CAP guidelines [2,4]. All data were uploaded to a secure Google Cloud Platform (GCP, Menlo Park, CA, USA) environment for processing and analysis.

### 2.2. Model Implementation

Both TITAN (Boston, MA, USA) [20] and CHIEF (Boston, MA, USA) [21] were used at their published architectures and pre-trained weights, without task-specific fine-tuning. Slide-level TITAN features for TCGA-BRCA were obtained from the publicly released TCGA_TITAN_features.pkl file in the MahmoodLab/TITAN Hugging Face repository; no in-house WSI preprocessing was performed for TITAN. Slide-level CHIEF features were extracted in-house from the TCGA-BRCA diagnostic WSIs using the Trident pipeline, an open-source, model-agnostic WSI processing framework that standardizes tissue segmentation, tile coordinate extraction, patch-level encoding, and slide-level aggregation across pathology foundation models. We ran Trident with its recommended configuration for the CHIEF slide encoder: tissue segmentation by the default HEST deep-learning segmenter (confidence threshold: 0.5); exhaustive tiling at a 10× equivalent magnification with 256 × 256-pixel patches; per-patch encoding by the CTransPath patch encoder; and slide-level aggregation by the CHIEF slide-level model. The output is one 768-dimensional embedding per slide. No explicit stain normalization is applied in either pipeline; ImageNet mean/standard-deviation normalization is applied at the patch-encoder input. The number of tiles per slide is determined by each slide’s tissue area and is not pre-specified; for TCGA-BRCA, the typical range is on the order of several hundred to several thousand tiles per slide. Both pipelines produce 768-dimensional slide-level vectors that were used directly as inputs to the downstream linear-probe head described in Section 2.3.

### 2.3. Experimental Design

All data partitioning was performed strictly at the TCGA case (patient) level. Unique patient identifiers (submitter_id) were first partitioned into training (50%), validation (25%), and testing (25%) sets by stratified random sampling on the patient-level biomarker label. When multiple WSIs were available for a given patient, all slides for that patient were assigned exclusively to a single split, with no overlap of patient identifiers across splits (verified at runtime via disjointness assertions). The 50/25/25 design was selected to balance training sample size with independently held-out validation and testing cohorts. For ER and PR, results were interpreted as binary outcomes (positive or negative) based on clinical reporting standards (≥1% nuclear staining considered positive) [1,27]. For HER2 IHC, scores of 0 and 1+ were considered negative, 2+ was considered equivocal, and 3+ was considered positive; FISH results served as ground truth for equivocal (2+) cases, consistent with ASCO/CAP guidelines [2,4]. Logistic regression on model embeddings was used to generate probability scores for threshold-dependent metrics; the L2 regularization strength C was selected on the validation set by log-loss minimization over a 45-point logarithmically-spaced grid. To reduce sensitivity to any single partition, the patient-level splitting procedure was repeated across 10 independent random seeds (seeds 42, 1, 2, …, 9). For each seed, the linear-probe pipeline was re-trained on the training split, hyperparameters were selected on the validation split, and metrics were evaluated on the held-out testing split. Metrics are reported as the mean across seeds together with percentile-based 95% confidence intervals (see Section 2.4).

### 2.4. Performance Metrics

Model performance was evaluated using accuracy, balanced accuracy, weighted F1-score, AUROC, AUPRC, sensitivity, specificity, positive predictive value (PPV), and negative predictive value (NPV). AUROC was calculated by plotting sensitivity against 1-specificity across all classification thresholds. AUPRC was calculated as the average precision across all thresholds and is interpreted as a precision−recall summary across thresholds rather than as the precision at any specific operating point. Sensitivity, specificity, PPV, and NPV are reported at the default 0.5 decision threshold. AUPRC was selected as a co-primary metric because of the following reasons: (1) it summarizes precision−recall behavior across thresholds, which is informative for clinical workflows that may operate at different thresholds; (2) it penalizes false positives more heavily than AUROC in imbalanced datasets; and (3) its random-classifier baseline equals class prevalence, enabling direct interpretation relative to the prior probability of disease. All metrics are reported as the mean across 10 independent patient-level random partitions, together with percentile-based 95% confidence intervals defined by the 2.5th and 97.5th percentiles across the 10 seeds. For the seed-42 reference run, we additionally report bootstrap standard deviations for AUROC and AUPRC (1000 resamples of the held-out test set). Between-model comparisons are presented with overlapping 95% CIs where applicable; formal pairwise tests (e.g., DeLong test for AUROC, paired bootstrap for AUPRC) on independent cohorts are planned for the external validation work.

## 3. Results

### 3.1. Overall Model Performance

Table 1 presents the comparative performance of TITAN and CHIEF across the three breast cancer biomarkers under the patient-level 10-seed evaluation, reporting mean with percentile-based 95% confidence intervals across seeds. TITAN and CHIEF achieved comparable AUROC values for ER (TITAN: 0.885 [95% CI: 0.848, 0.921] vs. CHIEF: 0.877 [0.831, 0.914]) and overlapping AUPRC values for ER and PR. For HER2, both models achieved AUROC values of approximately 0.71–0.74 with AUPRC values of approximately 0.41–0.45—clearly above the prevalence-based random baseline (approximately 0.211) but well below the values expected for clinically reliable threshold-specific operation (see Section 4.1 for interpretation).

AUPRC analysis clarifies the differences between biomarkers. For ER, TITAN achieved an AUPRC of 0.954 [95% CI: 0.940, 0.964] and CHIEF achieved an AUPRC of 0.955 [0.938, 0.969], indicating excellent precision−recall performance across thresholds; at the default 0.5 operating point, TITAN reported a PPV of 0.898 [0.870, 0.927] and CHIEF reported a PPV of 0.885 [0.860, 0.919]. For PR, TITAN achieved an AUPRC of 0.868 [0.850, 0.886] and CHIEF achieved an AUPRC of 0.864 [0.820, 0.907]; default-threshold PPV was 0.807 [0.783, 0.843] and 0.785 [0.759, 0.804], respectively. For HER2, AUPRC was 0.453 [0.347, 0.527] for TITAN and 0.406 [0.332, 0.455] for CHIEF—approximately 2-fold above the prevalence baseline of 0.211—but the very low default-threshold sensitivity (0.066–0.083) and wide PPV confidence intervals indicate that, at the default 0.5 threshold and without operating-point calibration, neither model produces well-calibrated HER2 predictions.

### 3.2. Estrogen Receptor (ER) Performance

**Figure 1:**
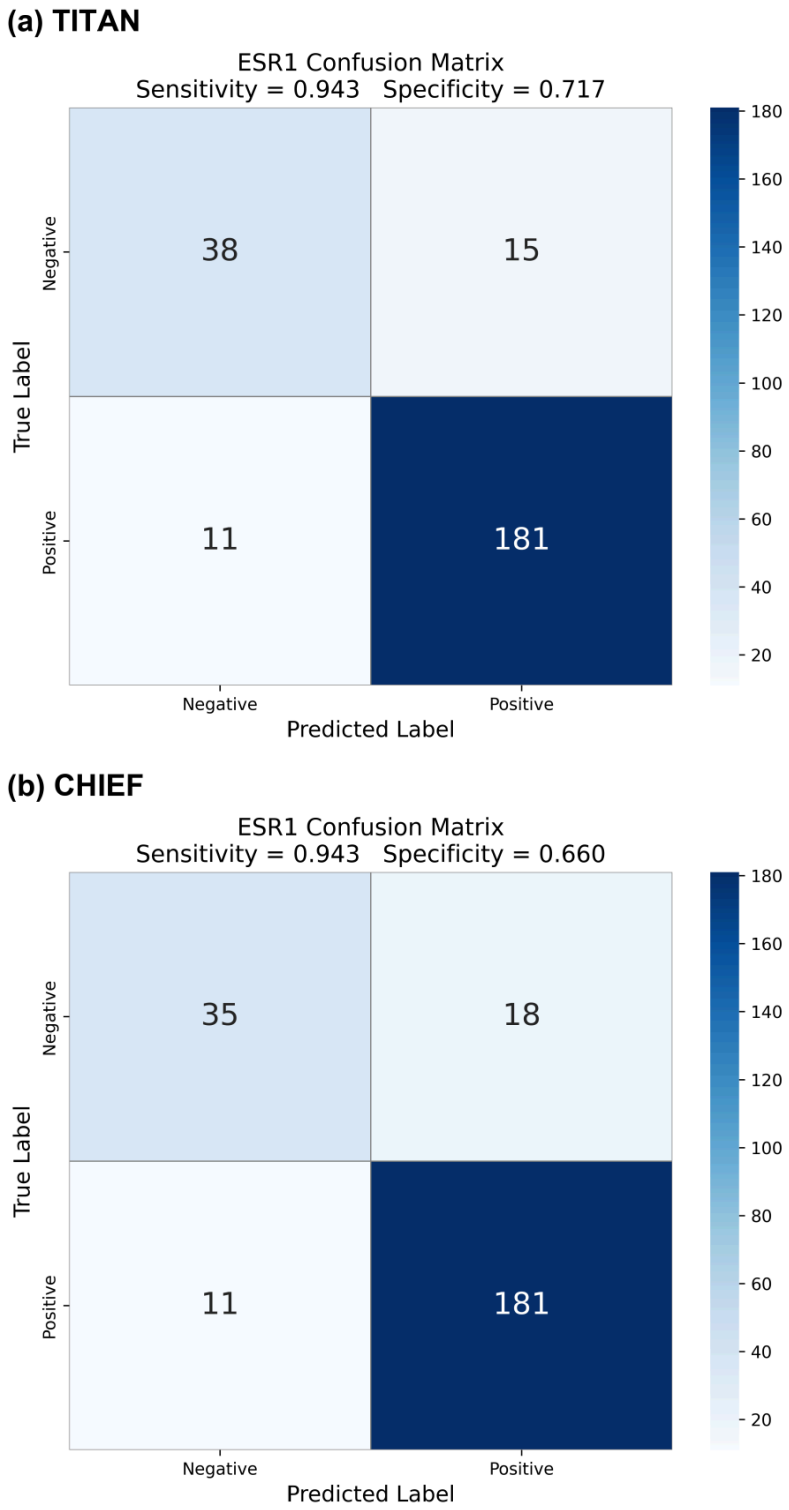
ESR1 confusion matrices. (**a**) TITAN: sensitivity = 0.943, specificity = 0.717. (**b**) CHIEF: sensitivity = 0.943, specificity = 0.660. ESR1—estrogen receptor status. Values shown reflect the patient-level seed-42 reference run at the default 0.5 operating threshold; aggregated 10-seed metrics with 95% CIs are reported in Table 1.

**Figure 2:**
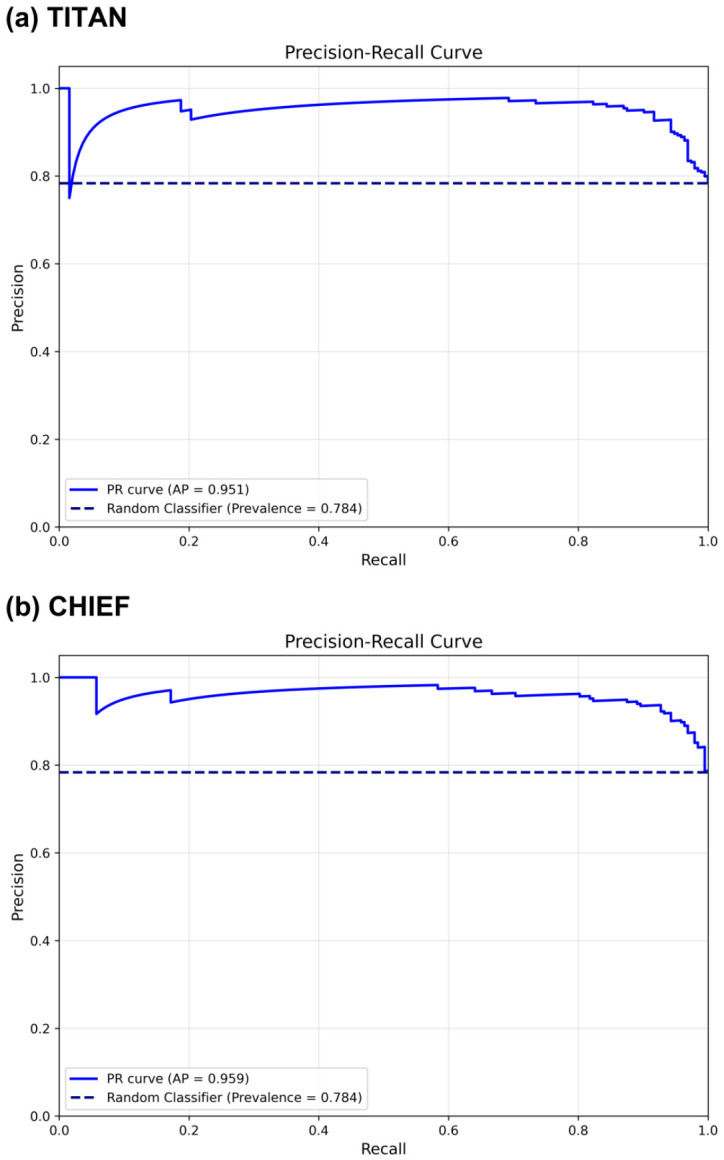
ESR1 precision−recall curves. (**a**) TITAN: AP = 0.951. (**b**) CHIEF: AP = 0.960. ESR1—estrogen receptor status. Values shown reflect the patient-level seed-42 reference run; the 10-seed AUPRC mean with 95% CIs is reported in Table 1.

**Figure 3:**
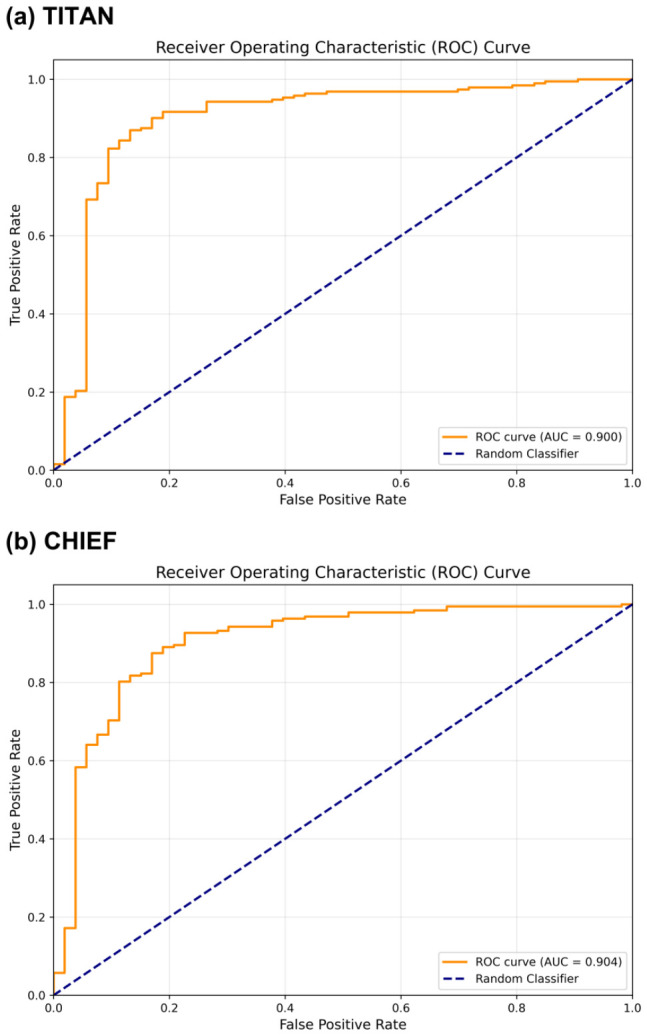
ESR1 ROC curves. (**a**) TITAN: AUC = 0.900. (**b**) CHIEF: AUC = 0.904. ESR1—estrogen receptor status. Values shown reflect the patient-level seed-42 reference run; the 10-seed AUROC mean with 95% CIs is reported in Table 1.

Both models achieved strong ER classification performance under the patient-level 10-seed evaluation. At the default 0.5 operating threshold and on the seed-42 reference run (Figure 1: ESR1 confusion matrices), TITAN achieved a sensitivity of 0.943 and a specificity of 0.717; the precision−recall curve (Figure 2) maintained high precision through most of the recall range, with an AUPRC of 0.954 (95% CI: 0.940, 0.964) over the 10 seeds. CHIEF achieved a sensitivity of 0.943 and a specificity of 0.660 (seed 42), with an AUPRC of 0.955 (95% CI: 0.938, 0.969). The ROC curves (Figure 3) show that TITAN achieved an AUROC of 0.885 [0.848, 0.921] and CHIEF achieved an AUROC of 0.877 [0.831, 0.914]. The default-threshold PPV was 0.898 [0.870, 0.927] for TITAN and 0.885 [0.860, 0.919] for CHIEF, indicating that ER-positive calls from either model are correct in approximately 88–90% of cases under this retrospective evaluation. The two models’ 95% CIs overlap on all reported ER metrics, so we report them as comparable rather than ranking them.

### 3.3. Progesterone Receptor (PR) Performance

**Figure 4:**
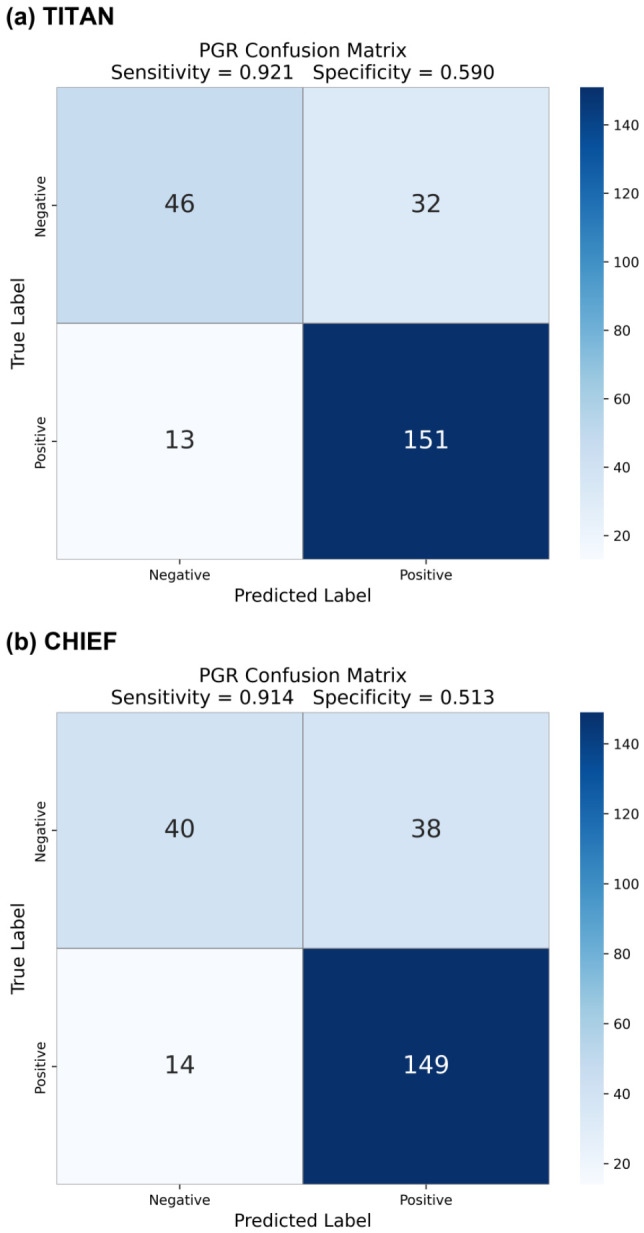
PGR confusion matrices. (**a**) TITAN: sensitivity = 0.921, specificity = 0.590. (**b**) CHIEF: sensitivity = 0.914, specificity = 0.513. PGR—progesterone receptor status. Values shown reflect the patient-level seed-42 reference run at the default 0.5 operating threshold; aggregated 10-seed metrics with 95% CIs are reported in Table 1.

**Figure 5:**
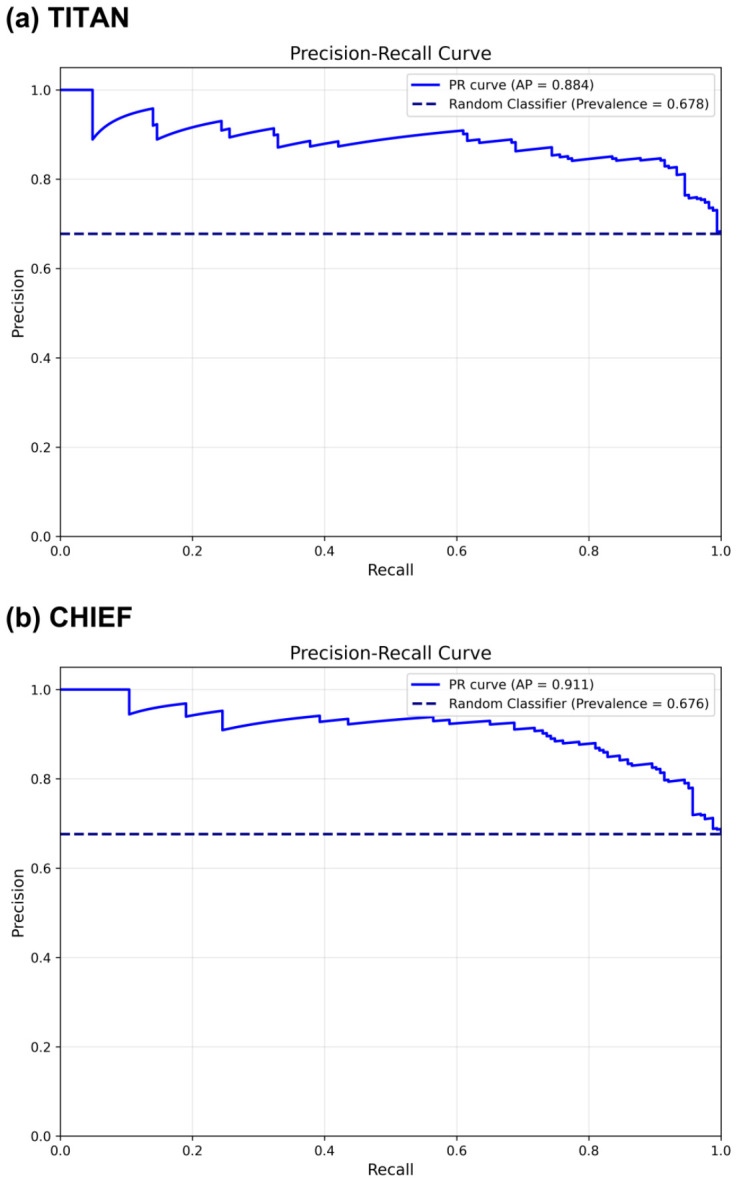
PGR precision−recall curves. (**a**) TITAN: AP = 0.884. (**b**) CHIEF: AP = 0.911. PGR—progesterone receptor status. Values shown reflect the patient-level seed-42 reference run; the 10-seed AUPRC mean with 95% CIs is reported in Table 1.

**Figure 6:**
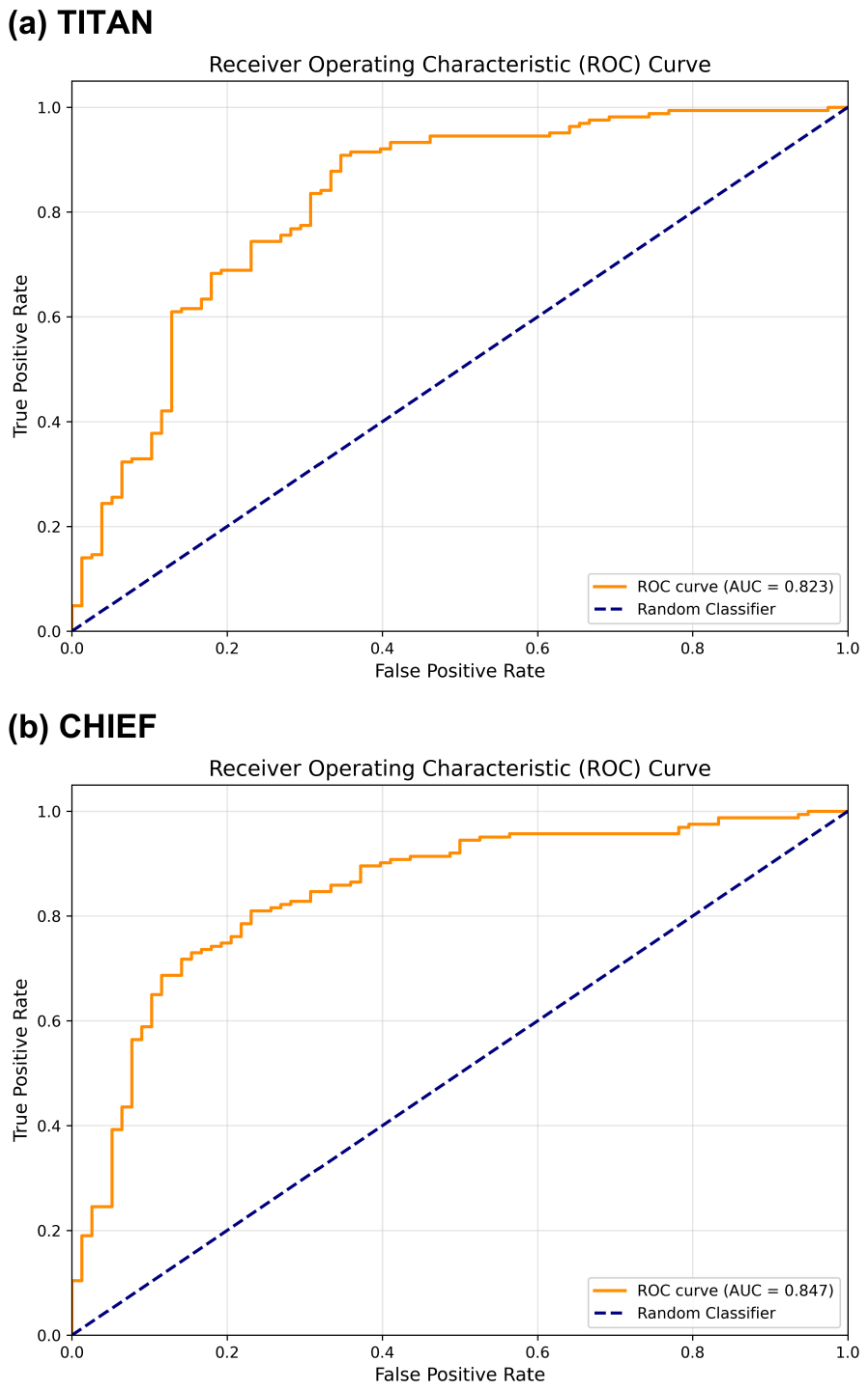
PGR ROC curves. (**a**) TITAN: AUC = 0.823. (**b**) CHIEF: AUC = 0.847. PGR—progesterone receptor status. Values shown reflect the patient-level seed-42 reference run; the 10-seed AUROC mean with 95% CIs is reported in Table 1.

PR classification performance was moderate. On the seed-42 reference run, TITAN achieved a sensitivity of 0.921 and a specificity of 0.590 (Figure 4: PGR confusion matrices); the precision−recall curve (Figure 5) had an AUPRC of 0.868 (10-seed 95% CI: 0.850, 0.886). CHIEF achieved a sensitivity of 0.914 and a specificity of 0.513 (seed 42), with an AUPRC of 0.864 (95% CI: 0.820, 0.907). The ROC curves (Figure 6) show that TITAN achieved an AUROC of 0.799 [0.766, 0.826] and CHIEF achieved an AUROC of 0.791 [0.747, 0.841]. Default-threshold PPV was 0.807 [0.783, 0.843] for TITAN and 0.785 [0.759, 0.804] for CHIEF, indicating that roughly one in five PR-positive calls would be incorrect at the default operating point—consistent with PR’s well-documented context-dependent expression patterns and its weaker H&E morphologic correlate compared with ER.

### 3.4. HER2 (ERBB2) Performance

**Figure 7:**
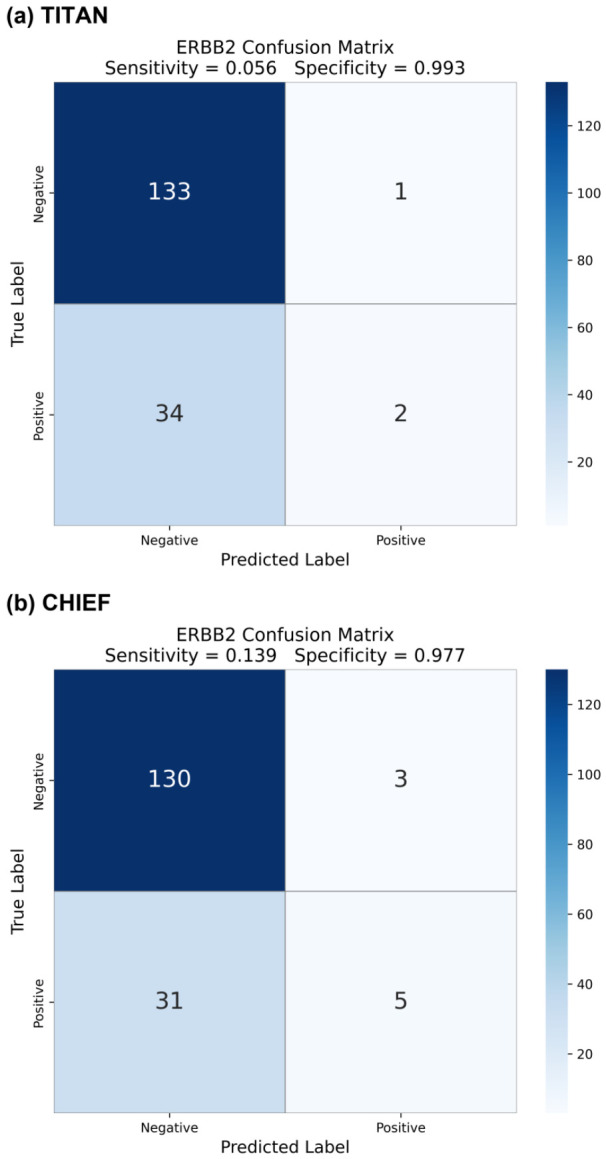
ERBB2 confusion matrices. (**a**) TITAN: sensitivity = 0.056, specificity = 0.993. (**b**) CHIEF: sensitivity = 0.139, specificity = 0.977. ERBB2—HER2 receptor status. Values shown reflect the patient-level seed-42 reference run at the default 0.5 operating threshold; aggregated 10-seed metrics with 95% CIs are reported in Table 1.

**Figure 8:**
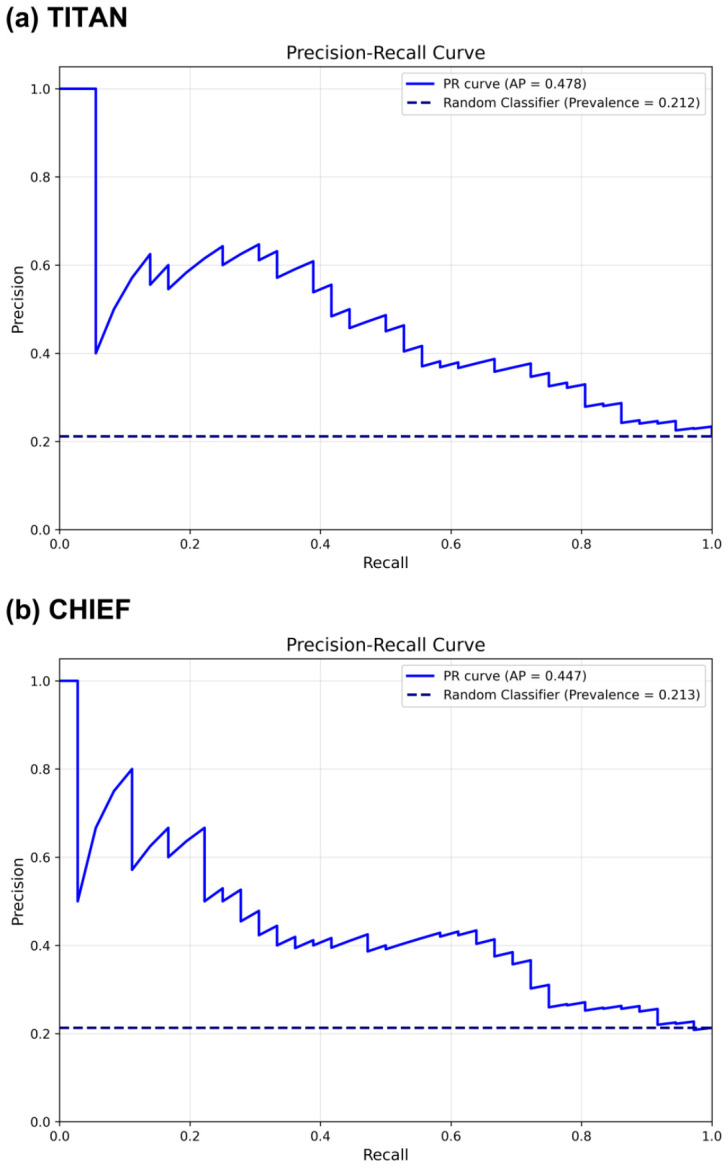
ERBB2 precision−recall curves. (**a**) TITAN: AP = 0.478. (**b**) CHIEF: AP = 0.447. ERBB2—HER2 receptor status. Values shown reflect the patient-level seed-42 reference run; the 10-seed AUPRC mean with 95% CIs is reported in Table 1.

**Figure 9:**
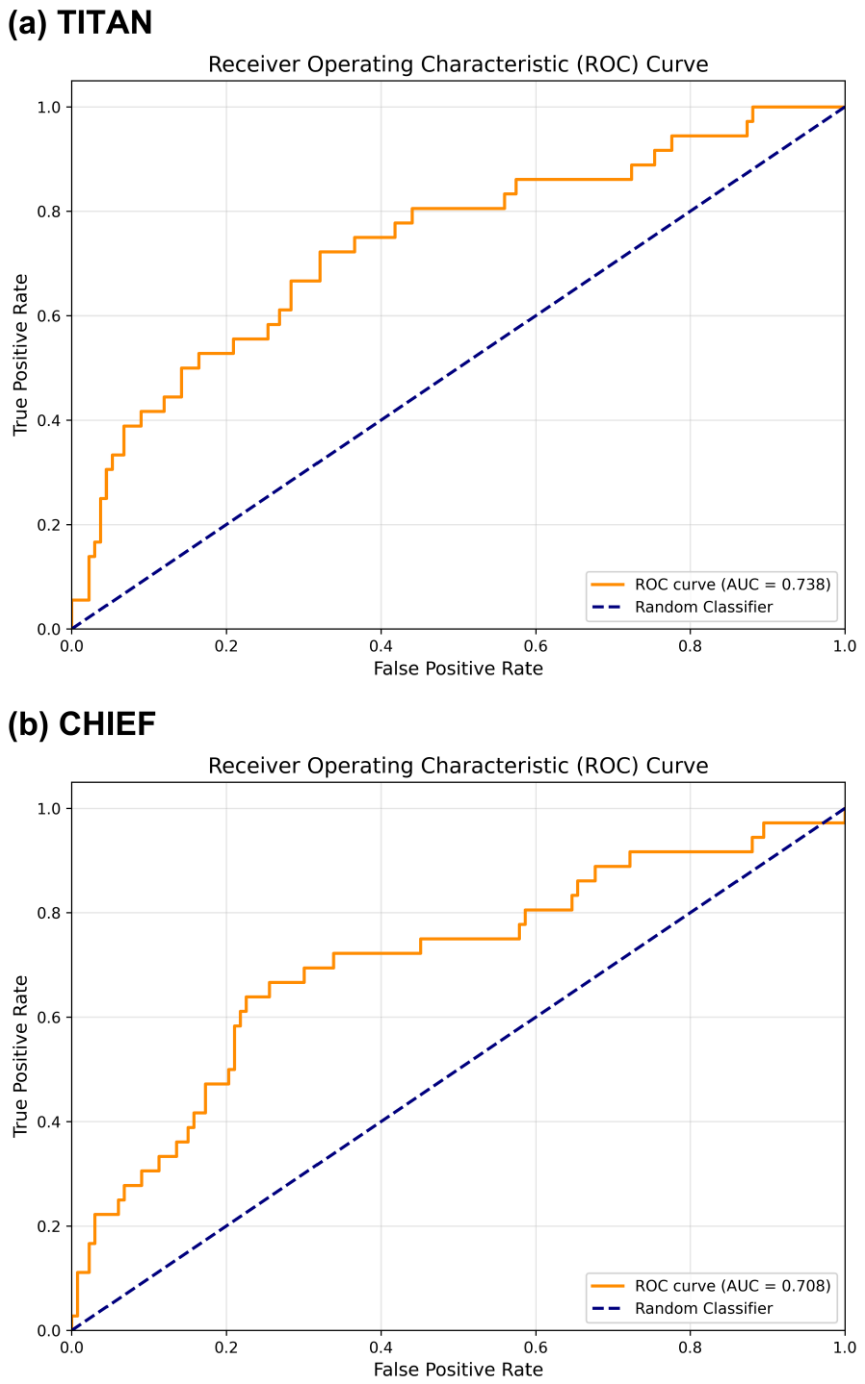
ERBB2 ROC curves. (**a**) TITAN: AUC = 0.738. (**b**) CHIEF: AUC = 0.708. ERBB2—HER2 receptor status. Values shown reflect the patient-level seed-42 reference run; the 10-seed AUROC mean with 95% CIs is reported in Table 1.

HER2 classification under the default 0.5 decision threshold and without explicit class-imbalance handling produced very low default-threshold sensitivity for both models. On the seed-42 reference run, TITAN achieved a sensitivity of 0.056 and a specificity of 0.993 (Figure 7a); CHIEF achieved a sensitivity of 0.139 and a specificity of 0.977 (Figure 7b). The precision−recall curves (Figure 8) show a 10-seed AUPRC of 0.453 (95% CI: 0.347, 0.527) for TITAN and 0.406 (95% CI: 0.332, 0.455) for CHIEF, both well above the prevalence-based random baseline (approximately 0.211). The ROC curves (Figure 9) show an AUROC of 0.743 [0.697, 0.799] for TITAN and 0.706 [0.645, 0.754] for CHIEF, indicating that some discriminative signal is recoverable from the foundation-model embeddings. However, at the default 0.5 threshold, both models predict almost exclusively the majority (negative) class, producing wide and unstable PPV confidence intervals across seeds (TITAN PPV: 0.446 [0.000, 0.931]; CHIEF PPV: 0.498 [0.075, 0.916]). This pattern reflects the following: (i) the class imbalance of HER2 in TCGA-BRCA (approximately 21% positive prevalence at the slide level), (ii) the use of a default 0.5 decision threshold without operating-point calibration, and (iii) the substantial morphologic heterogeneity of HER2-positive disease [28]—not a fundamental absence of morphologic signal.

## 4. Discussion

### 4.1. Biomarker-Specific Findings and Clinical Implications

#### 4.1.1. Estrogen Receptor: A Strong Morphologic Correlate with Clinical Feasibility

Under the patient-level 10-seed evaluation, TITAN achieved an AUROC of 0.885 [95% CI: 0.848, 0.921] and an AUPRC of 0.954 [0.940, 0.964] for ER. The AUPRC value summarizes precision−recall performance across thresholds; it is not a statement of threshold-specific precision. At the default 0.5 operating point, the positive predictive value (PPV) was 0.898 [0.870, 0.927], indicating that approximately 88–90% of ER-positive calls under this retrospective setup are correct. This aligns with the well-established biological correlation between ER positivity and consistent H&E features: tubule formation, lower nuclear grade, and organized stromal patterns [29,30]. The combination of stable cross-threshold precision−recall behavior and high default-threshold PPV under retrospective evaluation supports the hypothesis that H&E-based ER inference from either model could in future be evaluated as the following: (1) a candidate triage aid prior to IHC ordering, (2) a quality-control or discordance-flagging aid against existing IHC results, (3) a tool for resource-constrained settings where IHC is not routinely available, or (4) inference of ER status in archival cases lacking IHC data [7,10,31]. CHIEF achieved comparable results (AUROC: 0.877 [0.831, 0.914], AUPRC: 0.955 [0.938, 0.969], PPV: 0.885 [0.860, 0.919]). H&E-based ER inference from either model demonstrates strong discriminative performance under retrospective evaluation, warranting prospective validation prior to any clinical application. External validation on independent institutional cohorts, threshold and calibration analysis, and CLIA regulatory compliance review are required before any clinical deployment.

#### 4.1.2. Progesterone Receptor: Moderate Performance with a Supportive Evidence Role

Both models showed moderate performance for PR under the patient-level 10-seed evaluation: TITAN achieved an AUROC of 0.799 [0.766, 0.826] and an AUPRC of 0.868 [0.850, 0.886]; CHIEF achieved an AUROC of 0.791 [0.747, 0.841] and an AUPRC of 0.864 [0.820, 0.907]. This lower performance compared to ER reflects PR’s biological heterogeneity—PR expression is context-dependent, variably correlated with ER and less consistently manifested in H&E morphology [32]. AUPRC summarizes precision−recall performance across thresholds rather than threshold-specific precision; default-threshold PPV is 0.807 [0.783, 0.843] for TITAN and 0.785 [0.759, 0.804] for CHIEF, corresponding to approximately 19–21% false-positive calls at the chosen 0.5 operating point. This performance is appropriate for supportive workflow roles such as triage or quality control under prospective validation, but not for stand-alone clinical decision-making; IHC confirmation remains mandatory for PR [32]. The 95% CIs for TITAN and CHIEF overlap on AUROC and AUPRC, so we describe their PR performance as comparable rather than ranking them.

#### 4.1.3. HER2: Limited Discrimination Under the Default Operating Point

Under the linear-probe setup with a default 0.5 decision threshold and without explicit class-imbalance handling, neither TITAN nor CHIEF produces well-calibrated HER2 predictions. AUROC is 0.743 [0.697, 0.799] for TITAN and 0.706 [0.645, 0.754] for CHIEF—well above chance—and AUPRC is 0.453 [0.347, 0.527] and 0.406 [0.332, 0.455], respectively, approximately 2-fold above the prevalence baseline of 0.211. These values indicate that some discriminative signal is recoverable from the foundation-model embeddings. However, default-threshold sensitivity is very low (approximately 0.07–0.08) and default-threshold PPV is highly unstable across seeds. This pattern reflects the following: (i) HER2’s substantial class imbalance in TCGA-BRCA, (ii) the use of a default 0.5 decision threshold without operating-point calibration, and (iii) the substantial morphologic heterogeneity of HER2-positive disease [32,33] rather than a categorical absence of morphologic signal. Threshold calibration (e.g., Youden’s J on the validation set), class-balancing strategies, explicit modeling of HER2 morphologic heterogeneity, and multimodal approaches that incorporate genomic or proteomic data remain promising directions for future work; HER2 prediction from H&E alone in the present setup is not appropriate for clinical decision-making.

### 4.2. Model Comparison and Architecture Insights

The pathology foundation model landscape is rapidly evolving; as of 2025, approximately 30 patch-level and 8 WSI-level models have been described [18,19]. Among the prominent open-source options, patch-level encoders such as UNI and CONCH have demonstrated strong performance on tile classification tasks, while multimodal models including PLIP and BioViL leverage vision-language alignment. However, for WSI-level biomarker inference—which requires integrating information across the entire slide, WSI-level models are most relevant. We selected TITAN and CHIEF for complementary reasons: TITAN, because it represents the current state-of-the-art among open-source WSI FMs, with benchmarking demonstrating superiority over UNI and CONCH on multiple tasks [17,18] and CHIEF, because it is the smallest FM across major open-source options, requiring fewer computational resources and achieving shorter inference times [21]. CHIEF’s compact footprint opens possibilities for integration into low-cost digital pathology devices, resource-limited settings, or even direct embedding into scanning microscopes—a deployment pathway not currently feasible with larger models.

TITAN was pre-trained on 335,645 WSIs from diverse neoplastic, infectious, and inflammatory cases at Mass General Brigham using both visual self-supervised learning and vision-language alignment with over 182,000 pathology reports [20]. Its superior AUPRC across ER and PR likely reflects richer contextual representations from this large-scale multimodal pretraining. CHIEF leverages two complementary pretraining strategies—unsupervised tile-level feature learning and weakly supervised whole-slide pattern recognition—developed on 60,530 WSIs spanning 19 anatomical sites [21]. CHIEF’s competitive accuracy for HER2 and its favorable deployment profile make it an important benchmark for pragmatic clinical integration scenarios. These findings emphasize that model selection should consider not only aggregate performance but also calibration, interpretability, computational feasibility, and the specific clinical application [15,18].

### 4.3. Establishing AUPRC as Essential for Clinical Utility Assessment

A central methodological contribution of this work is to clarify how AUPRC should be reported and interpreted alongside AUROC in pathology-AI clinical-translation studies. First, AUPRC summarizes precision−recall behavior across all thresholds and is distinct from threshold-specific precision (positive predictive value, PPV). A clinician deploying an AI model at a chosen operating point needs to know the threshold-specific PPV at that operating point; AUROC alone, which integrates over all thresholds and over both classes, does not directly answer that question. AUPRC’s threshold-integrated precision−recall summary, together with explicitly reported PPV at the chosen operating point, jointly address this need. Second, AUPRC’s random-classifier baseline equals class prevalence, making performance immediately interpretable in the context of disease frequency. For HER2 at approximately 21% slide-level prevalence, an AUPRC of 0.41–0.45 indicates that the models add only modest precision−recall improvement over a prevalence-only classifier. Third, AUPRC is more sensitive than AUROC to improvements in detecting the minority class, which typically represents the clinically actionable group. The HER2 example in this study is illustrative: an AUROC of 0.71–0.74 appears marginally functional, while an AUPRC of 0.41–0.45 together with a default-threshold sensitivity of approximately 0.07–0.08 reveals that, under the default 0.5 threshold and without operating-point calibration, neither model produces clinically acceptable HER2 predictions. Reporting AUROC alone, without AUPRC and threshold-specific PPV, would have presented HER2 performance as more reassuring than it is at the evaluated operating point. We therefore recommend that pathology-AI publications and regulatory submissions report AUPRC alongside AUROC, together with sensitivity, specificity, and PPV at the chosen operating point, as a minimum standard.

### 4.4. Technical Challenges and Methodological Approach

An alternative approach to a frozen encoder with linear probing (logistic regression) FM evaluation would be to train a custom model on the TCGA-BRCA dataset with its biomarker labels. However, this approach faces significant challenges: IHC and FISH ground truth labels in TCGA were generated across multiple institutions with variable protocols, creating label noise that can propagate through and bias a trained model. Training on imperfectly annotated data risks encoding systematic errors from label heterogeneity rather than true morphologic signal. Our approach—evaluating pre-trained FMs in a frozen encoder with linear probing (logistic regression) setting—avoids training-time contamination by annotation noise, is immediately reproducible by other groups using the same publicly available models and directly tests whether the morphologic features learned during broad pre-training generalize to biomarker inference without task-specific fine-tuning. The key practical implication of this framework is that institutions can deploy these models without the infrastructure required for local model training, making FM-based biomarker inference accessible to a broader range of healthcare settings. Additionally, IHC interpretation thresholds have evolved over time (e.g., refined HER2-low and HER2-ultralow categories), and training on historical labels may not capture current clinical standards [19].

### 4.5. Interpretability and Trust

A critical limitation of this initial validation is the absence of interpretability analysis. For clinical translation, pathologists require not only accurate predictions but also interpretable rationales aligned with known biological mechanisms and histologic correlates [15,16]. Prior studies have demonstrated the value of attention visualization, region-level attribution analysis, and systematic comparison of model focus areas with pathologist-identified features [30]. Future work must incorporate these interpretability approaches—including Testing with Concept Activation Vectors (TCAVs) and saliency analysis—to provide transparent, actionable insights for clinical users [27].

### 4.6. Robustness, Generalizability, and Operational Considerations

This study utilized the TCGA-BRCA dataset, an aggregate of cases from multiple institutions, scanners, staining protocols, and patient populations—a feature that likely supports generalizability relative to single-institution datasets and may explain why performance approximates original published metrics for each model [20,21]. However, pre-analytical variables including fixation time, tissue processing, section thickness, and scanning parameters can substantially affect WSI appearance and model performance [5,14]. External validation on independent institutional cohorts is necessary before clinical deployment. Beyond diagnostic accuracy, clinical integration also requires addressing inference latency, computational requirements, failure mode characterization, uncertainty quantification, and integration with laboratory information systems [7,10,33]. Production deployment would further require rigorous validation pipelines, version control, model monitoring, and mechanisms for continuous learning as guidelines evolve [16,33].

### 4.7. Limitations

Several limitations must be acknowledged. First, the patient-level 50/25/25 split with 10 random repetitions on a single public dataset (TCGA-BRCA) provides limited evidence of generalization; external validation on independent institutional cohorts with diverse scanners and staining protocols is necessary and is a required next step before clinical translation [13,14]. Second, demographic and clinical variables were not analyzed, precluding fairness assessment across patient subgroups [12]. Third, all available histologic subtypes were combined; DCIS, IDC, ILC, mixed, and special-type cases were not modeled or analyzed separately, and subtype-stratified analysis is identified as a meaningful direction for future work. Fourth, HER2 predictions in the present study were evaluated at a default 0.5 decision threshold without operating-point or probability-calibration analysis; class-balancing and threshold-calibration strategies were not explored, so the reported HER2 sensitivity, specificity, and PPV should be interpreted as properties of the evaluated setup rather than as fundamental limits of morphology-based HER2 inference. Fifth, formal pairwise tests (e.g., DeLong test for AUROC, paired bootstrap for AUPRC) on independent cohorts are planned for the external validation work; in the present manuscript, between-model comparisons are presented with overlapping 95% CIs where applicable. Sixth, the study focuses only on slide-level binary classification without examining spatial heterogeneity or cases near decision boundaries [26]. Seventh, Ki-67, an important additional breast biomarker, was not evaluated [34]. Finally, interpretability analyses—necessary for understanding which histologic features drive predictions—are not yet available.

### 4.8. Future Directions

Immediate priorities include external validation on independent institutional cohorts to confirm the AUROC and AUPRC values reported here, with a unified preprocessing protocol across cohorts to ensure comparability. Operating-point and probability-calibration analyses—particularly for HER2—should determine whether the low default-threshold sensitivity reflects miscalibration that is correctable through threshold tuning or class-rebalancing, versus more fundamental limits of morphology-only HER2 inference that would motivate multimodal approaches (e.g., integration of genomic or proteomic features). Sensitivity analyses with alternative linear probes (e.g., linear SVM, ridge classifier) and non-linear heads (e.g., MLP, random forest), comprehensive multi-model benchmarking—including a controlled comparison of patch-level (UNI, CONCH, etc.) and slide-level (TITAN, CHIEF, GigaPath, PRISM, etc.) foundation models under a unified aggregation protocol—and subtype-stratified analyses are valuable directions for dedicated follow-up studies. Longer-term opportunities include multi-task learning approaches that jointly predict multiple biomarkers and integrate clinical context, uncertainty quantification to flag unreliable predictions, fine-tuning and few-shot strategies for rare subtypes, and prospective studies evaluating impact on turnaround time and clinical workflow [7,8,10,16,35,36]. Recent advances in pseudo-labelling and feature discrimination strategies for medical-imaging foundation models [37] also offer promising directions for label-efficient adaptation in the WSI biomarker context. Formal pairwise statistical comparisons (e.g., DeLong test for AUROC, paired bootstrap for AUPRC) are planned for the external validation cohort.

## 5. Conclusions

This study presents a comparative evaluation of two strategically selected open-source pathology foundation models—TITAN (a high-capacity vision-language WSI foundation model, approximately 48.5 M parameters) and CHIEF (a parameter-efficient WSI foundation model, approximately 1.2 M parameters)—for breast cancer biomarker inference from H&E-stained WSIs using publicly available TCGA-BRCA data accessed through the NCI Imaging Data Commons and Genomic Data Commons.

Under a patient-level 10-seed evaluation with percentile-based 95% confidence intervals, TITAN and CHIEF achieved AUROC values of 0.885/0.877 and AUPRC values of 0.954/0.955 for ER, AUROC values of 0.799/0.791 and AUPRC values of 0.868/0.864 for PR, and AUROC values of 0.743/0.706 and AUPRC values of 0.453/0.406 for HER2. The default-threshold PPV was 0.90/0.89 for ER, 0.81/0.79 for PR, and unstable for HER2.

A central methodological contribution is to clarify that AUPRC should be reported as a precision−recall summary across thresholds, alongside threshold-specific PPV at the chosen operating point, rather than as a substitute for the latter. For biomarkers where false-positive calls at the chosen operating point carry significant clinical or economic costs, reporting AUROC alone is insufficient. Reporting AUPRC alongside AUROC, together with sensitivity, specificity, and PPV at the chosen operating point, should be considered a minimum standard for pathology AI publications and regulatory submissions.

The findings of this study are hypothesis-generating. ER detection demonstrates strong discriminative performance under retrospective evaluation, warranting prospective validation, threshold and calibration analysis, and clinical-workflow study before any clinical application is considered. PR detection is moderate and would similarly require prospective validation prior to any clinical use. HER2 prediction at the default 0.5 threshold under the linear-probe setup evaluated here is not appropriate for clinical decision-making; threshold calibration, class-rebalancing, and multimodal extensions remain promising directions. External validation across independent multi-institutional cohorts, calibration analysis, and clinical-workflow integration are required before clinical deployment is considered. CHIEF’s substantially smaller parameter count makes it an interesting candidate for resource-constrained settings if its performance is confirmed externally, and frames the TITAN−CHIEF comparison as an accuracy-efficiency trade-off worth characterizing in subsequent work.

## Figures and Tables

**Table 1:** Comparative performance of TITAN and CHIEF models for breast biomarker detection. Values are reported as mean across 10 patient-level random partitions with percentile-based 95% confidence intervals in brackets, except for the random baseline row (theoretical reference).

Model	Biomarker	AUROC	AUPRC	Sensitivity	Specificity	PPV
TITAN	ER (ESR1)	0.885 [0.848, 0.921]	0.954 [0.940, 0.964]	0.953 [0.928, 0.968]	0.613 [0.494, 0.721]	0.898 [0.870, 0.927]
CHIEF	ER (ESR1)	0.877 [0.831, 0.914]	0.955 [0.938, 0.969]	0.948 [0.931, 0.963]	0.553 [0.459, 0.694]	0.885 [0.860, 0.919]
TITAN	PR (PGR)	0.799 [0.766, 0.826]	0.868 [0.850, 0.886]	0.920 [0.886, 0.959]	0.524 [0.437, 0.636]	0.807 [0.783, 0.843]
CHIEF	PR (PGR)	0.791 [0.747, 0.841]	0.864 [0.820, 0.907]	0.913 [0.879, 0.945]	0.462 [0.363, 0.528]	0.785 [0.759, 0.804]
TITAN	HER2 (ERBB2)	0.743 [0.697, 0.799]	0.453 [0.347, 0.527]	0.083 [0.000, 0.221]	0.979 [0.938, 1.000]	0.446 [0.000, 0.931]
CHIEF	HER2 (ERBB2)	0.706 [0.645, 0.754]	0.406 [0.332, 0.455]	0.066 [0.006, 0.137]	0.984 [0.973, 1.000]	0.498 [0.075, 0.916]
Random	HER2 baseline	0.500	~0.211 (prevalence)	--	--	--

## Data Availability

All data used in this study are publicly available through the National Institutes of Health Imaging Data Commons (https://imaging.datacommons.cancer.gov/) and Genomic Data Commons (https://gdc.cancer.gov/). The TITAN model is available at (https://github.com/mahmoodlab/TITAN) (accessed on 7 June 2026). The CHIEF model is available at (https://github.com/hms-dbmi/CHIEF) (accessed on 7 June 2026). Analysis code and detailed methodology are available from the corresponding author upon reasonable request.
